# Epidemiology of smoking among the employees of a medical university and strategies to decrease prevalence

**DOI:** 10.1186/1617-9625-12-S1-A3

**Published:** 2014-06-06

**Authors:** Karoly Viragh, Eniko Viragh

**Affiliations:** 1 Olive View UCLA Medical Center, Sylmar, Los Angeles, California, 91342, USA; 2 University of Medicine and Pharmacy, Targu Mures, 540139, Romania

## Background

The purpose of the study is to assess the prevalence of smoking among the employees of a medical university, to provide descriptive statistics of the actual situation, and to suggest university targeted policy changes in reducing smoking for the ultimate goal of a smoke-free campus.

## Materials and methods

A review of the medical files of all employees of the University of Medicine and Pharmacy, Targu Mures, Romania (UMF-TGM), was performed in the Occupational Health and Safety Office in July 2013. For each employee, the person’s age, gender, occupation, and smoking history (pack-years, reasons to start, reasons to quit) were recorded. Epidemiological data analysis was performed using Microsoft Excel and the data was organized in subgroups based on age, gender and occupation. The significance levels were assessed. The study was performed in concordance with university research policy.

## Results

UMF-TGM has 677 total employees, of which 6 had no information available. Of the 671 employees with information, the age range was 26-65 years with a median of 44 years. There were 218 smokers (32.0%, 1-to-56 pack-year range) and 453 non-smokers (68.0%), of which 406 never smoked and 47 were former smokers. There were 390 women (113 smokers, 29.0%) and 281 men (105 smokers, 37.0%). There were 375 health professionals, of which 295 were physicians (72 smokers, 24.0%), 51 were dentists (16 smokers, 31.0%), and 29 pharmacologists (6 smokers, 21.0%). The teaching faculty had 413 members (health professionals and non-health professionals), of which 108 were smokers (26.0%). The non-teaching university employees (technicians, administrative assistants, maintenance personnel) included 258 persons, of which 110 (43.0%) were smokers. Reasons to start smoking included the presence of smokers in the social environment, curiosity/boredom, and coolness. Reasons to quit smoking included health, money and social environment.

## Conclusions

Despite of major public health efforts, smoking remains one of the most important causes of morbidity and mortality. The prevalence rate of smoking at UMF-TGM is 32.0%, which was significantly higher in men and non-teaching employees than in women and teaching faculty. These rates are unacceptably high, given that a medical institution should set example of a smoke-free environment and healthy lifestyle. Current university smoking policy conforms to the national smoking policies; however, there are no targeted programs in place to reduce smoking. Therefore, initiatives will be presented to the university to increase awareness and assist current smokers in quitting.

